# Obituary: Dr. Margaretha Pangau-Adam

**DOI:** 10.1007/s00114-023-01878-1

**Published:** 2023-09-15

**Authors:** Matthias Waltert

**Affiliations:** Department of Conservation Biology, Faculty of Biology and Psychology, University of Göttingen, Bürgerstrasse 50, 37073 Göttingen, Germany

**Fig. 1:**
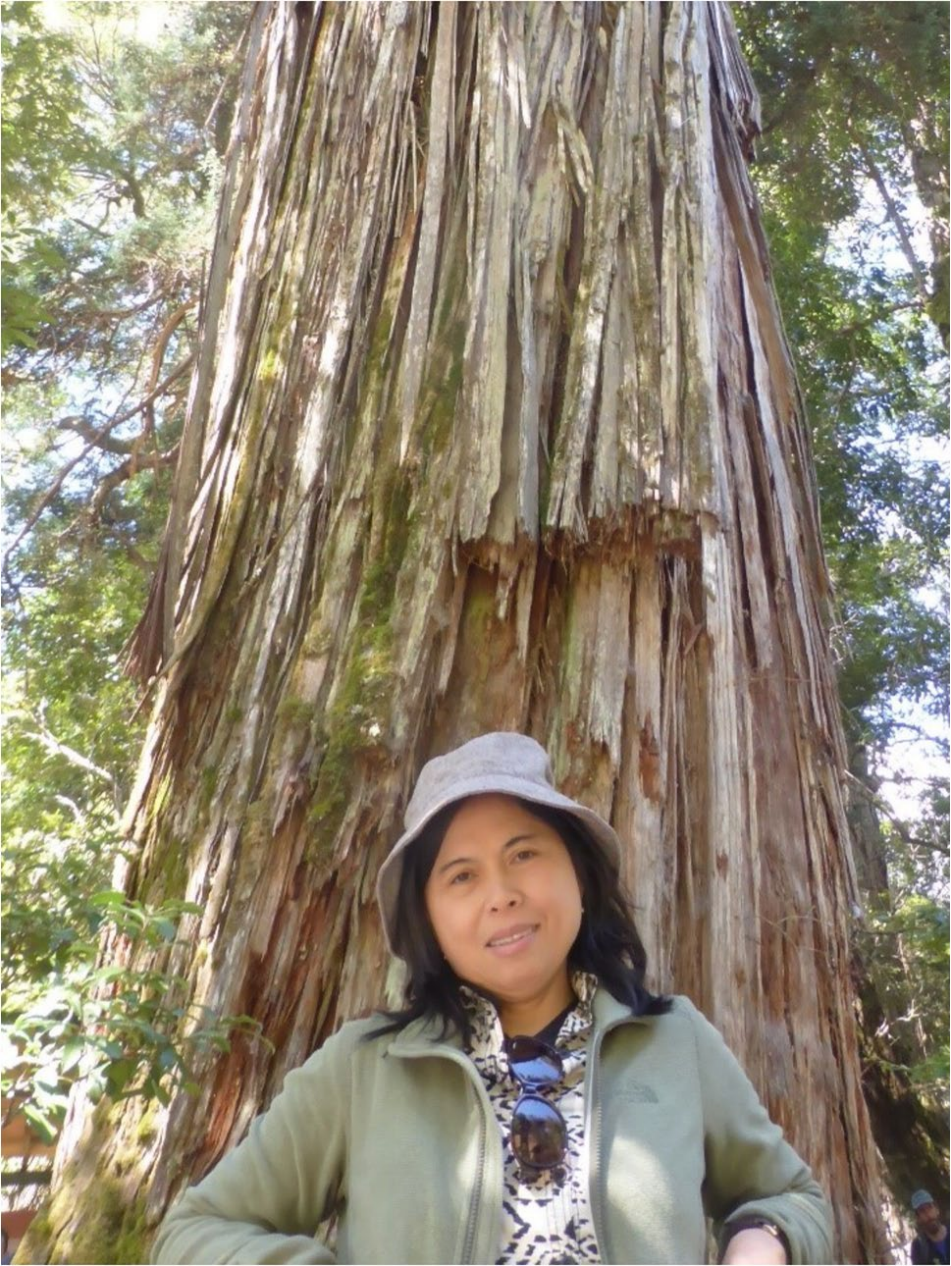
Margaretha Pangau-Adam

Dr. Margaretha Pangau-Adam, former student, postdoc, and guest researcher at the Department of Conservation Biology, University of Göttingen, Germany, and Senior Lecturer in Biology and Anthropology at Cenderawasih University, Papua, Indonesia, passed away in Mahembang, North Sulawesi, Indonesia (Fig. 1). She leaves behind her husband and her daughter.

Margaretha completed both her master’s and PhD degrees at Göttingen University to which she remained affiliated for 27 years (since 1996). Her career was that of a Tropical Ecologist and Wildlife Biologist with a focus on the Central and Eastern Indonesian archipelago and Papua. After her PhD on avian nest predation on Sulawesi, she shifted her focus towards human hunting and the conservation ecology of large birds such as the brush turkeys, cassowaries, and ground pigeons of Papua. She studied the diets of these large, ground-dwelling birds and documented effects of human hunting and habitat change on these enigmatic species. She was also a very important facilitator for research in the region, allowing for major discoveries, such as on the effects of biogeographic barriers on mammal abundance, the hunting skills of indigenous people, the systematic relationships of the highland wild dogs of Papua, and the systematic conservation planning on the island. Her most current research activities were on the role of the introduced rusa deer on Papua for local hunting and its potential effects on the vegetation and native vertebrates. She also bridged the continental gap for students, by facilitating exchange between Indonesian and German universities, organizing summer schools and taking students to the field. In addition, she provided wildlife population data to Göttingen university students so that they could improve their analytical and modelling skills during their winter course work.

Margaretha was well connected with internationally active organizations such as the German Academic Exchange Service DAAD and the Deutsche Gesellschaft für Internationale Zusammenarbeit GIZ. She was a Research Ambassador for DAAD and also worked for GIZ’s Forests and Climate Change Programme FORCLIME in Papua. As a conservationist, she was well regarded by all her contacts, allowing her to build an important network between people, and was able to convince decision-makers at all societal levels. As a trustworthy, honest, and deeply religious person, she had best relationships with local communities. She appreciated, and even loved, the hard work in the field and was an inspiring optimist. Her sudden and unexpected death at a young age is hitting everyone who knew her very hard.

Our team offers our deepest condolences for the passing of Dr. Margaretha Pangau Adam. Margaretha, beristirahat dalam damai! Margaretha, rest in peace!

